# The Role of Anterior Vaginal Prolapse in Co-Existent Underactive Overactive Bladder Syndrome—A Retrospective Cohort Study

**DOI:** 10.3390/jcm14020600

**Published:** 2025-01-17

**Authors:** Yoav Baruch, Marta Barba, Alice Cola, Matteo Frigerio

**Affiliations:** 1 Urogynecology and Pelvic Floor Unit, Department of Obstetrics and Gynecology, Tel Aviv Medical Center, Faculty of Medicine, Tel Aviv University, Tel Aviv-Yafo 6997801, Israel; 2 Department of Obstetrics and Gynecology, ASST Monza, San Gerardo Hospital, University Milano-Bicocca, 20900 Monza, Italyalice.cola1@gmail.com (A.C.); frigerio86@gmail.com (M.F.)

**Keywords:** coexistent detrusor overactivity–underactivity, CUOB, pelvic organ prolapses, underactive bladder, urodynamics

## Abstract

**Background:** CUOB (co-existent underactive overactive bladder) syndrome is a clinical entity that embraces storage and emptying symptoms, not strictly correlated with urodynamic findings. We assessed the differences between patients diagnosed with CUOB with/without cystocele. **Methods:** The study group was allocated from 2000 women who underwent urodynamic studies between 2008 and 2016. The demographic and clinical data of 369 patients with complaints consistent with CUOB were retrieved. The study group was subdivided using the Pelvic Organ Prolapse Quantification System. The International Consultation on Incontinence Questionnaire Short Form (ICIQ-UI SF) was used to quantify LUTS severity. **Results:** A total of 185 women had no or grade I cystocele (group 1), and 185 had grade II or III cystocele (group 2). No difference in mean age was computed. Patients from group 1 had a higher BMI (27 vs. 25, *p* = 0.02). Risk factors for prolapse, such as parity (1.7 vs. 2.1, *p* = 0.001) and maximal birthweight (3460 g vs. 3612 g, *p* = 0.049), were higher in group 2. Pelvic Organ Prolapse symptoms were 4.5 times more frequent in group 2 [n = 36/185 (19.5%) vs. n = 162/184 (88%) *p* < 0.001]. The rate of stress (70.8% vs. 55.4%, *p* = 0.002) and urge (64.9% vs. 50%, *p* = 0.04), urinary incontinence, and ICIQ-UI-SF scores (8 vs. 5, *p* < 0.001) were higher in group 1. Qmax measured lower in group 2 (17 vs. 15 mL/s, *p* = 0.008). Detrusor pressure at maximum flow was identical (24 cm H_2_O). The Bladder Contractility Index (BCI) was higher in group 1 (108 vs. 96.5, *p* = 0.017), and weak contraction (BCI < 100) was more common in group 2 (73/185; 39.5% vs. 95/184; 52.7%, *p* = 0.011). **Conclusions:** Based on our results, we assume that CUOB could be further subdivided based on its association with cystocele. The effect of prolapse repair in women with CUOB and cystocele remains to be evaluated in order to afford better counseling in the future.

## 1. Introduction

Patients with lower urinary tract symptoms (LUTS) are traditionally characterized as having either storage symptoms, such as urgency and urinary incontinence, or voiding symptoms, such as abnormally slow or incomplete micturition. Sometimes, patients can present with both [1,2,3]. LUTS are common in women after menopause [4,5] and, based on clinical manifestation, may be due to an overactive bladder (OAB) or an underactive bladder (UAB), classified as storage, voiding, or dysfunction, respectively. Based on urodynamic tests, these two entities relate to detrusor overactivity (DO) or underactivity (DU) [6].

The uroflow of healthy, neurologically intact women demonstrates that as the void begins, a rapid upsurge has an abundant peak flow rate, declines quickly with voiding times typically under 60 s, and reveals scant residuals. With voiding dysfunction, the flow curve can be flattened and prolonged, displaying suspended time to peak flow and a low or interrupted peak or flow rate, such as that which occurs with bladder outlet obstruction or detrusor underactivity, allowing for a large residual volume [7].

The co-existence of emptying and storage symptoms in one patient has recently acquired the acronym CUOB (co-existent underactive overactive bladder) [8,9]. CUOB is a clinical entity that embraces confluent LUTS irrespective of etiology and pathophysiology and is majorly correlated with urodynamic findings consistent with detrusor overactivity–underactivity (DOU). CUOB (irrespective of urodynamic evidence of DO and DU) is now considered a real clinical syndrome that differs from single OAB and UAB and is not necessarily a combination of OAB and UAB. CUOB may be caused by a variety of disorders, namely POP, neural decompensation, ischemia, fibrosis, and bladder outlet obstruction [5,9]. CUOB is thought to be multifactorial, with aging believed to play a major role due to cellular damage. The etiopathogenetic process, prevalence, and related urodynamic findings remain to be elucidated.

Recently, it was shown that 18.8% of women who presented with pelvic organ prolapse (POP) symptoms could be classified as CUOB and that the degree of observed proportionate concordance between CUOB and urodynamic findings suggestive of coexisting detrusor overactivity–underactivity (DOU) was as high as 75.8% [10]. CUOB is thought to be related, amongst other things, to increased urethral resistance, decreased detrusor competence, or both [10,11]. Otherwise, CUOB remains an emerging entity that needs to be better categorized.

The aim of this analysis was to evaluate the clinical and urodynamic findings of patients with CUOB related to the presence or absence of a significant cystocele.

## 2. Materials and Methods

This is a retrospective cohort study conducted at a tertiary university-affiliated medical center (San Gerardo Hospital, Monza, Italy). The study group was allocated from patients (n = 2000 women) who underwent urodynamic studies between 2008 and 2016 for Pelvic Floor Dysfunction (PFD) [10]. Of these, 369 women had complaints consistent with CUOB, and 1671 did not demonstrate CUOB.

A clinical interview was undertaken to inquire about LUTS, including urgency (OAB), urge urinary incontinence (UUI), stress urinary incontinence (SUI), voiding symptoms (VS), and bulging symptoms [12]. The International Consultation on Incontinence Questionnaire Short Form (ICIQ-UI SF) was used to subjectively quantify the patients’ perception of LUTS severity [13].

A urogenital examination and a urodynamic assessment were performed by a trained urogynecologist, as previously described [14]. The gynecological examination included POP staging according to the Pelvic Organ Prolapse Quantification (POP-Q) staging system [15]. All patients underwent urodynamic evaluation according to the International Continence Society (ICS) Good Urodynamic Practices and Terms [16]. The reasons for urodynamic evaluation were as follows: SUI in 61.1%, OAB in 57.5%, and voiding dysfunction in 35.6% with some patients having more than one indication.

The following parameters were evaluated during the storage phase: bladder volume at first and strong desire to void, presence or absence of urgency with or without the presence of urine leakage, maximum filling pressure (PdetMax), and maximum bladder capacity as well as presence or absence of leak with Valsalva. Evidence of leakage during intra-abdominal pressure-increasing maneuvers in the absence of a detrusor contraction was recorded as urodynamic stress urinary incontinence (UD-SUI). During the voiding phase, the following parameters were noted: maximum flow, maximal detrusor pressure (Pdet) at opening and closing, Pdet at maximal flow (PdetQmax), and postvoid residual volume (PVR). Voiding dysfunction was defined—according to ICS—as abnormally slow and/or incomplete micturition, based on abnormally slow urine flow rates and/or abnormally high post-void residuals. Abnormally slow urine flow rate was defined as <12 mL/s while positive PVR (PPVR) volume was defined as post-micturition residual >100 mL. Detrusor underactivity (DU) was evaluated through the Bladder Contractility Index (BCI) proposed by Abrams [17]. BCI < 100 was considered indicative of DU.

This study was approved by the Institutional Review Board ASST Monza Ethical Committee. Approval Code: RE-PFDs: 11 February 2022

### Statistical Analyses

Categorical variables were summarized as frequency and percentage. Normally distributed continuous variables were reported as mean and standard deviation (SD). Skewed variables were reported as median and interquartile range. The Chi-square test and Fisher’s exact tests were applied to compare categorical variables. An independent sample *t*-test and Mann–Whitney test were used to compare continuous variables. All statistical tests were two-sided and a *p*-value of less than 0.05 was considered statistically significant. SPSS software (IBM SPSS software for Windows, version 28, IBM Corporation, Armonk, New York, NY, USA, 2021) was used for all statistical analyses.

## 3. Results

Based on the POP-Q, the rate of cystocele among women with CUOB was significantly higher than the rate computed among women who did not demonstrate CUOB (184/369; 49.9% vs. 671/1671; 40.2% *p* = 0.006).

Based on the POP-Q, 185 subjects (185/369; 50.1%) had either no cystocele (grade 0; n = 102) or (grade I cystocele (n = 83) (group 1), and 184 (184/369; 49.9%) subjects had grade II and III cystocele (group 2). There were no patients with stage IV cystocele. Age was comparable between groups (61.7 ± 13 for group 1 vs. 64.7 ± 10 for group 2; *p* = 0.14). Patients from group 1 had a higher BMI (27 kg/m2 for group 1 vs. 25 kg/m2 for group 2; *p* = 0.02). Risk factors for prolapse, such as parity and maximal neonatal birthweight, were higher in group 2 (1.7 vs. 2.1, *p* = 0.001; 3460 g vs. 3612 g, *p* = 0.049). (Table 1).

### LUTS, Prolapse, and Urodynamic Characteristics

POP symptoms were 4.5 more frequent in group 2 (n = 162/184; 88%) than in group 1 (n = 36/185; 19.5%, *p* < 0.001). Prolapse of other (non-anterior) compartments was twice to thrice more prevalent in group 2; *p* < 0.001 (Table 1).

The full POP parameters are detailed in Table 2.

The rate of stress (131/185; 70.8% vs. 102/185; 55.4%, *p* = 0.002) and urge incontinence (120/185; 64.9% vs. 92/184; 50%, *p* = 0.004) as well as ICIQ-UI SF scores (8 vs. 5, *p* < 0.001) were higher in group 1.

Qmax measured slightly, but significantly, higher in group 1 (17 mL/s vs. 15 mL/s, *p* = 0.008). Bladder Contractility Index (BCI) was higher in group 1 (108 vs. 96.5, *p* = 0.017), and weak contraction (BCI < 100) was more common in group 2 (73/185; 39.5% vs. 95/184; 52.7%, *p* = 0.011). Detrusor pressure at a maximum flow rate (Pdet-Qmax) was identical (24 cm H_2_O; *p* = 0.278) in both groups. The Schafer nomogram used to evaluate bladder contractility revealed that women in group 2 had weaker bladder activity (normal vs. weak, *p* = 0.003). The proportion of patients with low BCI and positive PVR was higher in group 2 compared to group 1 (97/184; 52.7% vs. 73/185; 39.5%, *p* = 0.011 and 45/184; 24.5% vs. 27/185, 14.6% *p* = 0.017, respectively) (Table 3).

## 4. Discussion

CUOB diagnostic criteria have not yet been fully established. The etiopathogenetic process underlying CUOB, although not fully comprehended, is mainly attributed to the detrusor’s instability or incompetence. Different hypotheses proposed include sparse denervation and afferent dysfunctions, detrusor activation inefficiency, ischemic-reperfusion injury, accumulation of fibrous intramuscular connective tissue in the detrusor muscle, and bladder outlet obstruction-induced hypertrophy and decompensation [4,18,19]. It was previously demonstrated that detrusor function impairment is directly related to the severity of anterior compartment prolapse, which may cause mechanical bladder outlet obstruction by urethral kinking leading ultimately to detrusor hypertrophy and loss of function [20,21,22]. The notion that CUOB is preponderantly related to mechanical outlet obstruction is strengthened by the fact that cystocele is significantly more prevalent among women lacking CUOB symptomatology (*p* = 0.006).

The objective of this study was to differentiate between the clinical characteristics and urodynamic indices in a large group of older women diagnosed with CUOB. Uro-gynecological assessment revealed that significant cystocele (stage II and III) was present in 50% of patients with CUOB.

According to previous studies, the prevalence of CUOB among women scheduled for prolapse repair approaches 20% and patients with CUOB endure a more severe anterior compartment prolapse [10,11]. Here, we show (Table 3) that, compared to women with no or small-scale anterior wall prolapse, a higher proportion of women with stage II and III cystocele had a low BCI (97/184; 52.7% vs. 73/185; 39.5% *p* = 0.011), weaker bladder contractility (131/184; 71.2% vs. 104/185; 56.2%, *p* = 0.003), and positive PVR (45/184; 24.5% vs. 27/185;14.6%, (0.017). Women with cystocele presented with the lower maximum flow 15 (9–19) vs. 17 (10–23), *p* = 0.008) and, based on Schafer’s and Schafer’s simplified nomogram, which serve to discriminate DU from BOO [8], gained lower scores (3 (3–4) vs. 4 (3–5), *p* = 0.01) and (1 (1–2) vs. 2 (1–2), *p* = 0.003, respectively).

Stress incontinence was present in 57.5% (212/369) and urge incontinence was present in 63.1% (233/369) of our patients with CUOB. Interestingly, SUI and UUI were less prevalent in women with stage II and III anterior wall prolapse than in women with no or negligible anterior wall prolapse (92/184; 50.0% vs. 120/185; 64.9% and 102/184; 55.4% vs. 131/185; 70.8%, *p* = 0.004 and 0.002, respectively). This is plausibly related to the bigger urethral resistance imposed by the prolapse. In women with CUOB, the presence of bulging symptoms may mitigate OAB symptomatology. Conversely, as previously suggested, untreated prolapse may lead to impaired bladder contractility, ultimately resulting in detrusor underactivity [10,11]. It has been suggested that similarities in urodynamics findings between CUOB and DO may indicate a continuum between the two entities, with DOU likely representing the most severe aspect of the detrusor’s inability to function properly in patients with CUOB [10,11]. Earlier studies showed that patients with DO and impaired contractility are older than patients with DO and preserved contractility and that they are more frequently present with urinary retention and voiding symptoms [22]. Altogether, older age and estrogen deprivation incite CUOB development subject to progressive degeneration caused by cellular damage and apoptosis. Here, we demonstrate, based on urodynamic testing, that CUOB with anterior wall prolapse is characterized by lower Qmax, higher PPVR, lower Schafer indices, low BCI, and weak contractility, thereby highlighting the effect of obstruction. It seems that in 50% of patients with CUOB, bladder obstruction plays a major role in causing impaired bladder contractility. In patients with no-cystocele, the etiopathogenesis leading to CUOB is related to variable age-associated morbidities, and, as such, CUOB with or without anterior vaginal prolapse should be considered as two different entities.

It was shown that POP repair led to a marked reduction in CUOB symptoms, with low opening detrusor pressure identified as the sole independent predictor of postoperative CUOB [10,11], and it has been assumed that the more the detrusor is damaged and the more its contractility dented, the less bladder function is likely to recover following POP repair. As such, when CUOB is consequent to bladder outlet obstruction due to prolapse, treatment of prolapse should be prioritized to avoid future complications. This is particularly important since medication that aimed to either augment contraction or reduce outlet resistance have restricted practice in female patients and that the treatment of vaginal wall prolapse either by pessary or surgery is free of complications in the vast majority of cases.

Both CUOB and DOU are related to OAB syndrome, urge incontinence, and voiding symptoms and incorporate characteristics of both bladder overactivity and bladder underactivity. Still, the diagnosis of CUOB relies on clinical features rather than on urodynamic tests [20,22]. Urodynamic tests, however, remain constructive for diagnosis and management.

Our study is the first analysis of women with CUOB differentiated on account of the presence or absence of significant anterior wall prolapse. Urodynamic indices and POP-Q point measurements provide pilot estimates that can contribute to future comparative studies involving such patients. The primary strength of this study is the large cohort of women diagnosed with CUOB. Limitations include the retrospective study design and the diversified indications that led to the urodynamic evaluation.

Our findings strengthen the notion that CUOB is partly incited by mechanical outlet obstruction, yet the full pathophysiological process linking CUOB and prolapse remains to be elucidated within the frame of broader research.

## 5. Conclusions

Based on these findings, we do think that CUOB needs to be further categorized. Although being a clinical condition urodynamic studies still have a valid role in the interpretation, management, and monitoring of CUOB. Contrary to the expected, the rate of DO among women with or without anterior wall prolapse was similar regardless of the observation that UUI and SUI were less prevalent in women with cystocele. It is postulated that the detrusor’s overactivity projected by urodynamics possibly reflects hypertrophy but is still below the threshold of domineering incontinence.

Q Max resulted slightly lower among women with prolapse due probably to mechanical obstruction. The clinical distinction between CUOB with or without outlet obstruction whether associated or not with disturbed contractility may be valuable before considering prolapse repair. Altogether, the classification and urodynamic parameters of CUOB await to be sorted and defined. More studies are needed in order to further uncover the role of anterior prolapse in CUOB and thereby expand counseling with the prospects of alleviating CUOB symptoms.

## Figures and Tables

**Table 1:** Population characteristics and lower urinary tract symptoms.

Patients with CUOB (n = 369)	No Cystocele or Grade 1 (n = 185)	Cystocele Grade 2\3 (n = 184)	*p*
Age (years)	61.7 ± 13.8	64.7 ± 10.1	0.14
BMI (kg/m^2^)	27 (23.9–32)	25 (22.1–26.6)	0.002
Parity (n)	1.71 ± 1.1	2.14 + 1	0.001
Mean Birthweight (g)	3464 ± 541	3612.7 ± 540	0.049
Urge urinary incontinence	131 (70.8%)	102 (55.4%)	0.002
Stress urinary incontinence	120 (64.9%)	92 (50.0%)	0.004
ICIQ-UI SF	8 (3–13)	5 (0–11)	<0.001
Qtip value	40 (25–50)	40 (20–50)	0.79
POP symptoms	36 (19.5%)	162 (88%)	<0.001
Some degree of apical prolapse	61 (33%)	171 (92.9%)	<0.001
Some degree of posterior prolapse	78 (42.2%)	151 (82.1%)	<0.001

Data are presented as mean ± standard deviation or count (percentage), median, and IQR (interquartile range). Non-continuous data as absolute frequency. Abbreviations: CUOB, co-existent underactive overactive bladder; BMI—Body Mass Index; ICIQ-UI SF—International Consultation on Incontinence Questionnaire—Urinary Incontinence Short Form; POP—Pelvic Organ Prolapse.

**Table 2:** POP-Q Parameters.

Patients with CUOB (n = 369)	No Cystocele or Grade 1 (n = 185)	Cystocele 2\3 (n = 184)	*p*
Point Aa	−3 (−3–−2)	0.5 (0–2)	<0.001
Point Ba	−3 (−3–−2)	1 (0–2)	<0.001
Point Ap	−3 (−3–−2)	−2 (−2–−1)	<0.001
Point Bp	−3 (−3–−2)	−2 (−2–−1)	<0.001
Point C	−7 (−8–−6)	−0.25 (−4–−1)	<0.001
Point D	−8 (−9–−7)	−4 (−6–−3)	<0.001
Point GH	3 (3–3.5)	3.5 (3–4)	<0.001
Point PB	3 (3–3)	3 (2.5–3)	<0.001
TVL	10 (9–10)	9 (9–10)	0.428
Apical Stage	0 (0–1)	2 (1–2)	<0.001
Posterior Stage	0 (0–1)	1 (1–2)	<0.001

Note: Data are presented as median and IQR (interquartile range). Abbreviations: POP-Q—Pelvic Organ Prolapse Quantification system (centimeters); GH—Genital Hiatus; PB—Perineal Body; TVL—Total Vaginal Length; CUOB, co-existent underactive overactive bladder;

**Table 3:** Urodynamic parameters in women with CUOB.

Patients with CUOB (n = 369)	No cystocele or Grade 1 (n = 185)	Cystocele 2\3 (n = 184)	*p*
1st desire to void	129 (88.5–184)	138 (102.2–191.7)	0.114
MCC Maximal Capacity	380 (329–444)	372 (320–447)	0.804
Pdet end filling phase cm H_2_O	8 (5–13.5)	8 (5–12)	0.878
Opening Detrusor pressure cm H_2_O	20 (13–28)	21 (13–31)	0.522
Closing Detrusor pressure cm H_2_O	19 (11–32)	22.5 (14–33)	0.072
Qmax (mL/s)	17 (10–23)	15 (9–19)	0.008
Pdet at Qmax	24 (15.5–33.5)	24 (18–35)	0.278
PVR (mL)	0 (0–58)	1 (0–98)	0.141
PVR (%)	0 (0–16.4)	0.4 (0–26.5)	0.148
PPVR	27 (14.6%)	45 (24.5%)	0.017
BCI < 100	73 (39.5%)	97 (52.7%)	0.011
weak contraction (BOO excluded).	68 (36.8%)	83 (45.1%)	0.103
Qmax < 12 and pdet < 10	7 (3.8%)	8 (4.3%)	0.784
U-SUI	92(49.7%)	78 (42.4%)	0.157
DO in UDS	74 (40%)	77 (41.8%)	0.641
Voiding Difficulty on UDS	104 (56.2%)	131 (71.2%)	0.003
DOU	31 (16.8%)	37 (20.1%)	0.406
Bladder Contractility Index	108 (79–142)	96.5 (79–124)	0.017
Schafer	4 (3–5)	3 (3–4)	0.01
Schafer simplified	2 (1–2)	1 (1–2)	0.003
SUI Severity	0 (0–1)	0 (0–1)	0.332

Note: Data are presented as mean ± standard deviation or count (percentage), median, and IQR (interquartile range) Non-continuous data as absolute frequency. Abbreviations: CUOBS—Co-existent Overactive Underactive Bladder Syndrome; SUI—stress urinary incontinence; Qmax—maximal flow rate mL\s; pDet at Qmax = Detrusor Pressure at Maximum Flow; DOU—Detrusor Overactive Underactive; BOO—Bladder Outlet Obstruction; BCI—Bladder Contractility Index; PVR = PostVoid Residual; PVR% = (PVR/MCC) × 100; MCC =Maximum Cystometric Capacity; PPVR = Positive PVR (PPVR) volume. U-SUI = Urodynamic Stress Urinary Incontinence. DO in UDS—Detrusor Overactivity.

## Data Availability

The data that support the findings of this study are not openly available due to reasons of sensitivity and are available from the corresponding author upon reasonable request.
